# Interactions between brown planthopper (*Nilaparvata lugens*) and salinity stressed rice (*Oryza sativa*) plant are cultivar-specific

**DOI:** 10.1038/s41598-020-64925-1

**Published:** 2020-05-15

**Authors:** Md Khairul Quais, Asim Munawar, Naved Ahmad Ansari, Wen-Wu Zhou, Zeng-Rong Zhu

**Affiliations:** 1 State Key Laboratory of Rice Biology, Ministry of Agriculture; Key Laboratory of Molecular Biology of Crop Pathogens and Insects; Institute of Insect Sciences, Zhejiang University, Hangzhou Zhejiang, China; 2 Senior Scientific Officer, Rice Farming Systems Division, Bangladesh Rice Research Institute, Gazipur, Bangladesh

**Keywords:** Salt, Plant stress responses, Herbivory

## Abstract

Salinity stress triggers changes in plant morphology, physiology and molecular responses which can subsequently influence plant-insect interactions; however, these consequences remain poorly understood. We analyzed plant biomass, insect population growth rates, feeding behaviors and plant gene expression to characterize the mechanisms of the underlying interactions between the rice plant and brown planthopper (BPH) under salinity stress. Plant bioassays showed that plant growth and vigor losses were higher in control and low salinity conditions compared to high salinity stressed TN1 (salt-planthopper susceptible cultivar) in response to BPH feeding. In contrast, the losses were higher in the high salinity treated TPX (salt-planthopper resistant cultivar). BPH population growth was reduced on TN1, but increased on TPX under high salinity condition compared to the control. This cultivar-specific effect was reflected in BPH feeding behaviors on the corresponding plants. Quantification of abscisic acid (ABA) and salicylic acid (SA) signaling transcripts indicated that salinity-induced down-regulation of ABA signaling increased SA-dependent defense in TN1. While, up-regulation of ABA related genes in salinity stressed TPX resulted in the decrease in SA-signaling genes. Thus, ABA and SA antagonism might be a key element in the interaction between BPH and salinity stress. Taken together, we concluded that plant-planthopper interactions are markedly shaped by salinity and might be cultivar specific.

## Introduction

Plants are often exposed to multiple abiotic and biotic stressors at the same time in nature. For example, they may need to adapt to soil affected by salinity, heavy metals or drought as well as to attacks by herbivores and pathogens. Abiotic and biotic stresses interact at the cellular level and reaction to a combination of stresses, however, is often unique and cannot be explained from studying these stresses individually^1–4^.

Worldwide, increased soil salinity has negative impacts on about 30% of irrigated and 6% of the total land area^5^. More than 397 million hectares of agricultural land in Africa, Asia, Australia and North and South America, have been affected by salinity^6^, with a monetary loss of 12 billion US$ in agricultural production^7^. Salinization has increased due to the redistribution of salts in the soil during the conversion of wetlands or forests into farmland. Although the salinization of the soil occurs most in dry and semi-dry areas, it has been reported in almost all climatic areas^8^. Better understanding of the plant adaptive mechanisms to cope with the abiotic as well as biotic stresses can help in the development of interventions. Salinity affects nutrient uptake, growth, ion homeostasis, general plant metabolism and water uptake in plants^9–11^. This stress can also alter the direct and indirect immune metabolites of the plant^12^. In addition, insect herbivory may be positively^13–15^, negatively^16–18^ or neutrally^19,20^ influenced by soil salinity. To date most studies have focused on direct effects of salinity stress on plants and herbivores, not so much on plant-herbivore interactions.

Insect feeding behavior is considered to have pivotal role in plant-herbivore interactions and feeding behavior is largely influenced by host plant nutrition^21^. Plants often enhance the production of sugars, sugar alcohols, and amino acids to counter low osmotic potentials in response to salinity stress resulting in increased nitrogenous compound in plant^22^. Rise in sugar concentrations in phloem exudates may contribute to increase feeding of phloem suckers^23^. However, some researchers have shown that changes in phloem sap viscosity due to altered sugars and solute concentrations can increase the difficulty for sucking herbivores to acquire nutrients^24,25^. Moreover, excessive Na^+^ and Cl^−^ ions in plants in response to salt stress can reduce palatability^26,27^. However, given that herbivores require sodium in their diets, as sodium can accumulates in plant tissues, they should become more attractive to herbivores, thus resulting in increased herbivore densities under high salinity^28^. In addition to excessive accumulation of ions, salinity can also induce more negative water potentials in plants^29^. These changes in plant water potentials can affect the ability of sap-sucking insects consuming xylem sap that can facilitate them to overcome the high osmotic potentials of the phloem sap to extract nitrogen-rich nutritional compounds from the host plant^30,31^. Taken together, these results indicate toward complex feeding activities of phloem suckers under salinity stress.

Besides changes to the nutritional quality of plants, salinity stress also compels the plant to fine-tune the network of hormonal signaling cascades. Plant responses during salinity stress are mainly regulated by the stress hormone, ABA^32^. ABA interacts with other hormones to promote stomatal closures, regulate the expression of stress responsive genes and proteins as well as the accumulation of compatible solutes under stress^33–35^. Moreover, it also affects plant resistance to pathogens and herbivores^30,36^. However, there is no general consensus on the effects of ABA on plant resistance since both positive and negative effects have been documented. Under combination of abiotic and biotic stresses, enhanced ABA production mostly promotes plant susceptibility to disease and herbivore attacks through suppression of the SA/JA/ethylene-regulated defenses^37–39^. In certain cases, ABA can have the opposite effects on plant defenses resulting in increased resistances^40–43^. In rice, salicylic acid (SA) signaling pathway plays a major role in responses to infestations by sucking insects such as the brown planthopper (BPH)^44–46^. Thus, analyzing the feeding behavior of the brown planthopper and evaluating changes in plant defense gene expression would obtain a better understanding of the impacts of salinity stress on plant-planthopper interactions.

In this study, we investigated the impacts of salinity stress on rice plant-brown planthopper interactions. As salinity-induced morphological and metabolic responses in different rice cultivars were differed significantly^47^, we adopted a broad approach by studying the impact of different rice cultivars on brown planthopper performance as well as the effects of BPH on plant performance under salinity stress. We analyzed the feeding behavior of BPH and gene expression linked to major plant signaling pathways as the possible mechanisms underlining the interaction between plant and herbivory under salinity stress.

## Results

### Effects of salinity on plant growth

**Figure 1:**
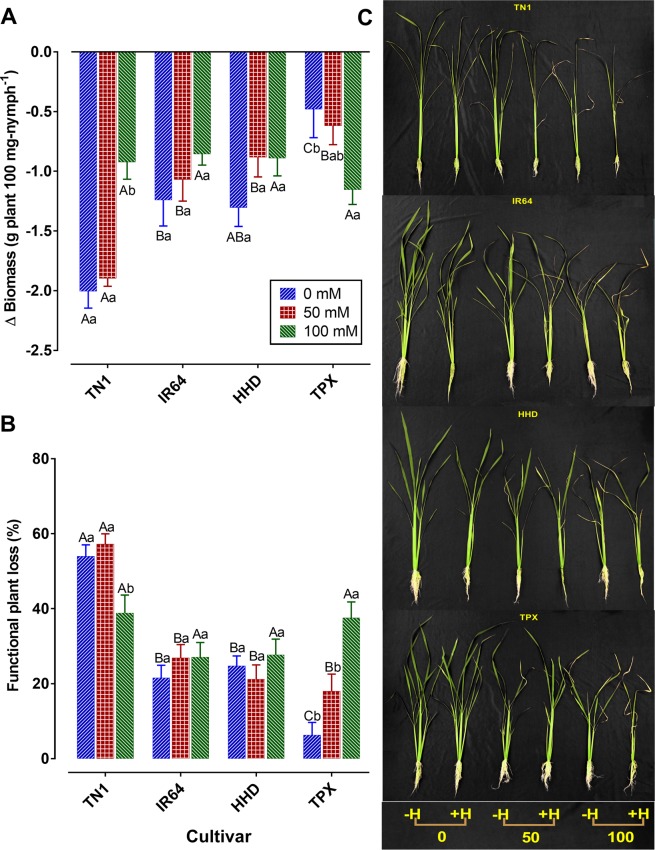
Effects of salt inputs and herbivory on rice cultivars. (**A**) Biomass difference (mean ± SE) between BPH infested and non-infested cultivars at different salinity levels. (**B**) Functional plant loss indices (mean ± SE) of plants infested with BPH at different salinity levels. Capital letters indicated the comparison among different cultivars within a given salinity level; Lower case letters indicated the comparison among different salinity levels within a given cultivar. Different letters indicate significant difference at *P* < 0.05. (**C**) Pictorial view of BPH infested (H^+^) and non-infested (H^−^) cultivars at different salinity levels. The figures were prepared and combined using Graphpad Prism version 8.3.1 for macOS (www.graphpad.com).

Plant growth parameters for BPH infested and non-infested plants under varying salinity levels are presented in Supplementary Table S1. The negative impact of BPH on plant biomass differed significantly among the salinity treatments (*F*_2, 108_ = 13.38, *P* < 0.001), rice cultivars (*F*_3, 108_ = 29.69, *P* < 0.001) and their interactions (*F*_6, 108_ = 16.26, *P* < 0.001) (Fig. 1A). The functional plant loss was also affected differently by salinity (*F*_2, 108_ = 3.53, *P* = 0.033) and cultivar (*F*_3, 108_ = 25.99, *P* < 0.001), and their interaction (*F*_6, 108_ = 6.79, *P* < 0.001) was significant (Fig. 1B). For TN1, plant biomass loss due to BPH infestation was significantly lower at high salinity level (100 mM) compared to those subjected to no (0 mM) and low salinity (50 mM). In contrast, the loss was significantly higher at high salinity compared to control in the case of TPX. However, there were no significant differences in biomass losses among different salinity levels in IR64 and HHD rice cultivars. In all cultivars, biomass loss was similar between BPH infested plants under control and low salt concentration. Plant biomass loss of TN1 cultivar was significantly higher than in other cultivars except for HHD at control (0 mM). At low salinity level, the loss in TN1 was significantly higher than other cultivars. However, it was similar among different cultivars under high salinity levels (Fig. 1A–C). The functional plant loss due to BPH infestation followed a similar pattern as biomass loss since plant functional loss of TN1 was significantly higher than HHD at control and in TPX, it was higher in low salinity compared to control treatment (Fig. 1B).

### Effects of salinity stress on BPH population growth

Population parameters of BPH were significantly affected by salinity stress on different rice cultivars (Table 1). Nymph numbers were significantly reduced on TN1 and IR64 plants at high salinity. In contrast, when the TPX plants at high salinity, it was significantly higher than that of the control. Salinity stress showed no effect on nymph numbers in the case of HHD. In the control, BPH produced higher offsprings on TN1 compared with IR64 and TPX cultivars and there was no significant difference in nymph numbers between TN1 and HHD. In plants exposed to low salinity, the planthopper produced more offspring on TN1 than on other cultivars. At high salinity, the nymph number was significantly higher on TN1 and HHD compared to TPX. However, for IR64 and TPX nymph numbers were similar. Salinity had no significant effects on hatching rate in the case of HHD and TPX, and only at high salinity the hatching rates in TN1 and IR64 were significantly different. In the control and low salinity, hatching rates were similar in all cultivars. While for TPX and HHD the rates were higher than TN1 at high salinity. The adult number followed a similar pattern as nymph since adult number of control treatment was significantly higher than that of high salinity on TN1 and at control treatment adult number was higher on TN1 compared to IR64 plants. Population growth index of control treatment was about 32, 15, and 18% higher than that of the high salinity level in TN1, IR64 and HHD, respectively, while the index of the control TPX plant decreased by 51%. BPH performed better on TN1, IR64 and TPX plants with lower salinity levels compared to the control. Irrespective of salinity, population index on TN1 was 35, 22, and 75% higher than that of IR64, HHD and TPX, respectively (Table 1).

**Table 1:** Changes in offspring number, hatching rate, and population growth of BPH reared on different rice cultivars under salinity stress.

Rice cultivar	NaCl level (mM)	Nymph number	Hatching rate	Adult number	Population growth index (N_1_/N_0_)
TN1	0	1284 ± 98Aab	0.91 ± 0.03Aa	1154 ± 70Aa	288.4
	50	1460 ± 138Aa	0.92 ± 0.02Aa	1333 ± 121Aa	333.3
	100	905 ± 64Ab	0.69 ± 0.04Bb	785 ± 51Ab	196.3
IR64	0	812 ± 100Bab	0.94 ± 0.01Aa	688 ± 68Bab	172.1
	50	1072 ± 79Ba	0.92 ± 0.03Aa	864 ± 46Ba	216.1
	100	727 ± 93ABb	0.82 ± 0.03ABb	587 ± 71ABb	146.7
HHD	0	1056 ± 58ABa	0.94 ± 0.01Aa	945 ± 37Aa	236.2
	50	943 ± 85Ba	0.88 ± 0.04Aa	838 ± 59Ba	209.5
	100	885 ± 73Aa	0.87 ± 0.01Aa	775 ± 66Aa	193.8
TPX	0	274 ± 31Ca	0.91 ± 0.03Aa	186 ± 27Ca	46.5
	50	347 ± 64Cab	0.82 ± 0.04Aa	258 ± 49Cab	64.5
	100	484 ± 56Bb	0.88 ± 0.01Aa	382 ± 50Bb	95.5
*F*-Cultivar (C)^a^		55.531***	0.989 ^ns^	86.993***	—
*F*-Salinity (S)^a^		6.200**	21.365***	9.098***	—
*F*-C × S^a^		4.375***	3.734**	6.438***	—

Values represent mean ± SE of five replicates. Capital letters indicated the comparison among different rice cultivars within a given salinity level; Lower case letters indicated the comparison among different salinity levels within a given cultivar. Different letters indicate significant difference at P < 0.05.

Nominator df = 3 (Cultivar), 2 (Salinity), 6 (C × S); denominator df = 48. ^a^ns = *P* > 0.05, * = *P* ≤ 0.05, ** = * P* ≤ 0.01, *** = *P* ≤ 0.001.

### Effects of salinity stress on BPH honeydew production

**Figure 2:**
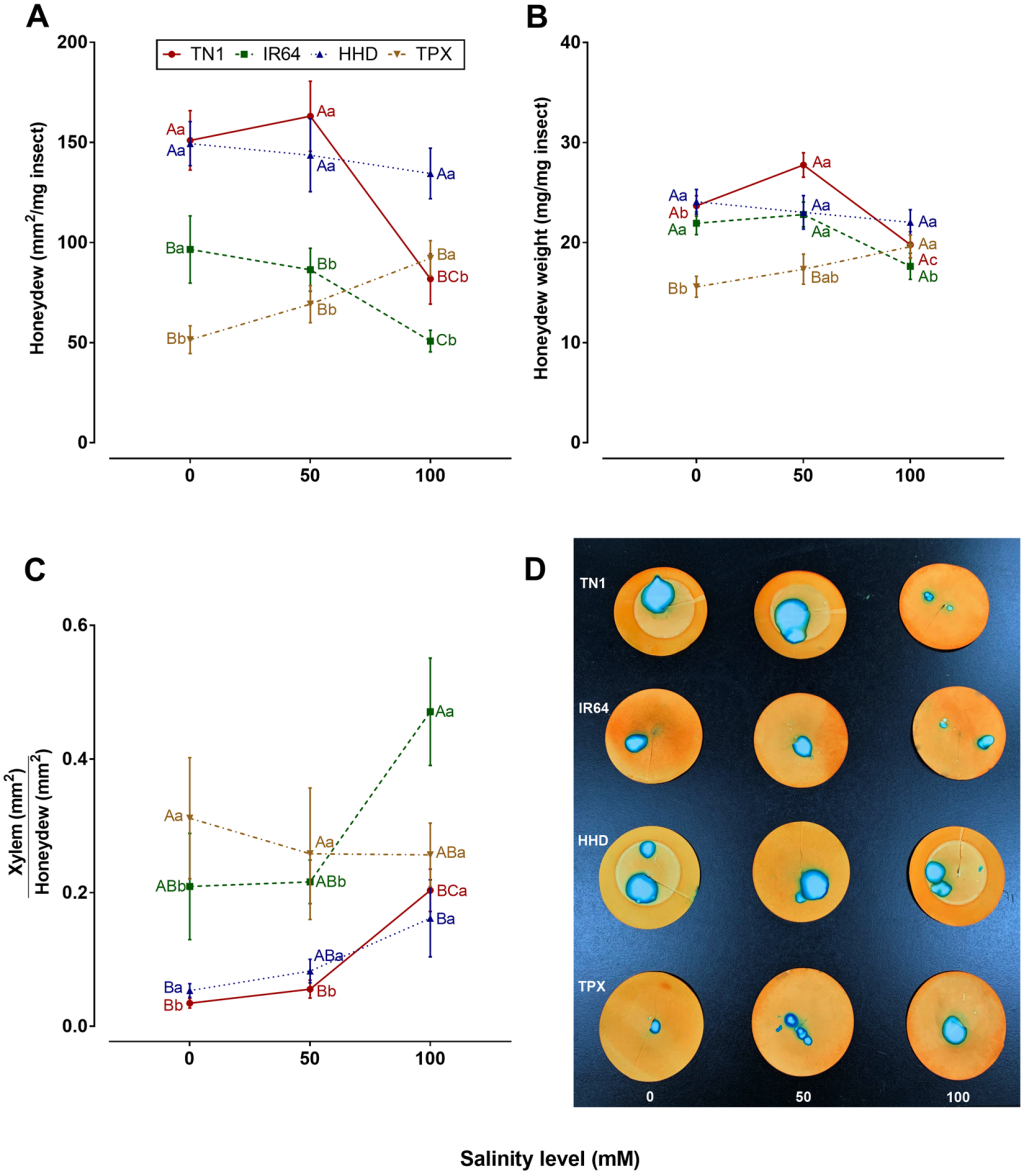
Responses of BPH to rice cultivars under salinity stress. Fitness responses included (**A**) honeydew production (mean ± SE) by BPH (Parafilm-sachet method), (**B**) honeydew area (mean ± SE) by BPH (Filter paper method), and (**C**) the proportion of honeydew that was derived from xylem (mean ± SE). Capital letters indicated the comparison among different cultivars within a given salinity level; Lower case letters indicated the comparison among different salinity levels within a given cultivar. Different letters indicate significant difference at *P* < 0.05. (**D**) Representative replicates showing amount of honeydew excreted by brown planthopper on filter paper. The figures were prepared and combined using Graphpad Prism version 8.3.1 for macOS (www.graphpad.com).

BPH honeydew production differed significantly among the salinity treatments (filter paper method: *F*_2, 108_ = 4.87, *P* = 0.009; parafilm sachet method: *F*_2, 348_ = 5.50, *P* = 0.004), rice cultivars (filter paper method: *F*_3, 108_ = 25.01, *P* < 0.001; parafilm sachet method: *F*_3, 348_ = 13.32, *P* < 0.001), and salinity × cultivar interaction (filter paper method: *F*_6, 108_ = 4.53, *P* < 0.001; parafilm sachet method: *F*_6, 348_ = 4.77, *P* < 0.001) (Fig. 2). In both methods, BPH excreted lower honeydew compare to control when they fed on TN1 and IR64 plants at high salinity level. The opposite trend was observed in TPX. Salinity showed no effect on honeydew production in the case of HHD. Among the cultivars, BPH produced more honeydew on TN1 indicating its susceptibility in the control and low salinity levels. Insects on TPX excreted the lowest amount of honeydew. The proportion of xylem-derived honeydew significantly affected by cultivars (*F*_3, 108_ = 11.78, *P* < 0.001), and salinity treatments (*F*_2, 108_ = 6.27, *P* = 0.003). There was no significant effect of salinity × cultivar interaction on xylem-derived honeydew excretion (*F*_6, 108_ = 1.48, *P* = 0.193) (Fig. 2). BPH produced more xylem-derived honeydew on TN1 and IR64, might be due to increased plant resistance in high salinity. Other cultivars showed no significant difference in response to salinity treatments. Planthopper on TPX and IR64 produced more xylem-derived honeydew than on TN1 and HHD in the control and low salinity. BPH excreted similar amount of xylem-derived honeydew on TPX and TN1 at high salinity.

### BPH probing and feeding behavior through EPG under salinity stress

**Figure 3:**
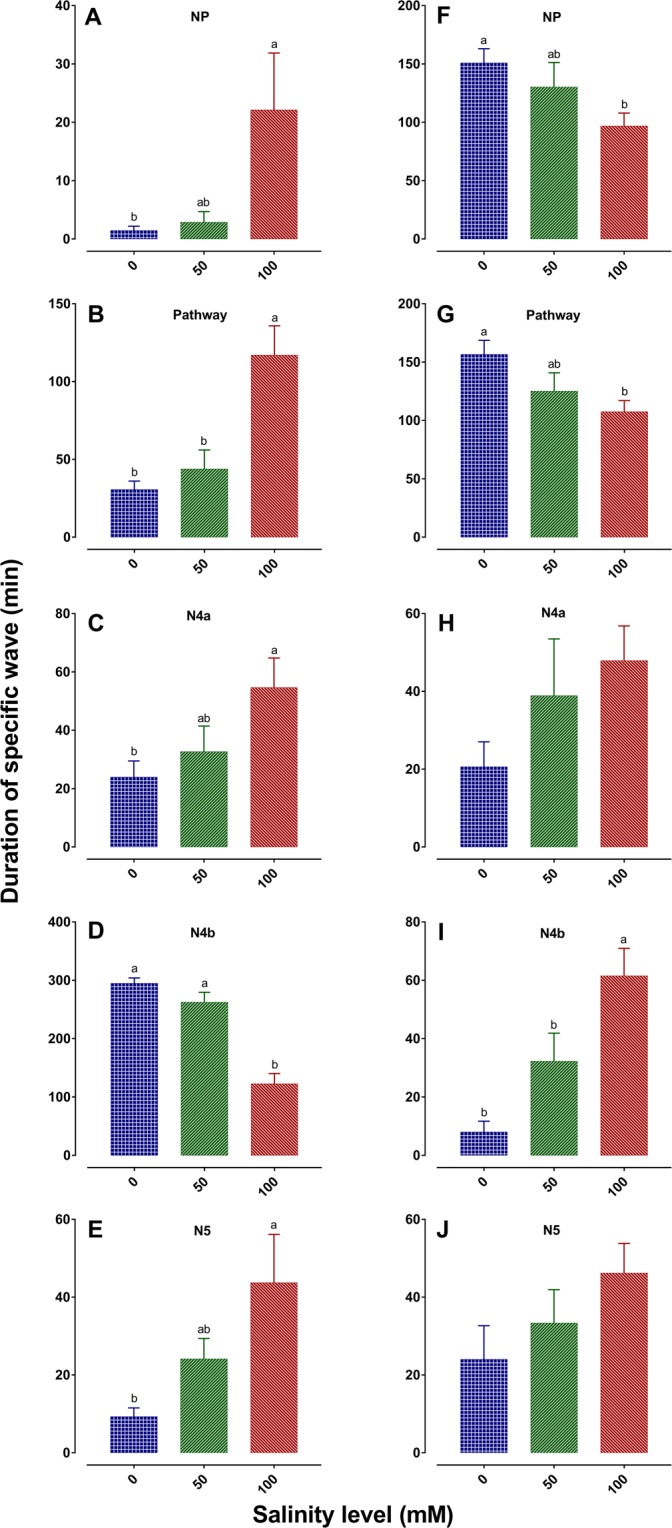
Feeding behaviors of BPH recorded by electrical penetration graph on TN1 (**A–E**) and TPX (**F–J**) plants at different salinity levels. (**A–J**) Duration of the different waveforms over a 6-h recording period; NP: Non-penetration, Pathway: Comprises of penetration initiation (N1), salivation and stylet movement (N2) and extracellular stylet activity near phloem region (N3), N4a: Phloem salivation, N4b: Phloem sap ingestion; N5: Xylem sap ingestion. Each bar represents the mean ± SE. Bars with different letters are significantly different (*α* = 0.05). Absence of letters indicates no significant difference among treatments. The data are averages of 10–13 recordings per treatment. The figures were prepared and combined using Graphpad Prism version 8.3.1 for macOS (www.graphpad.com).

The electrical penetration graph (EPG) recordings of BPH are presented in Fig. 3 and Supplementary Table S2. On TN1, all planthoppers started to penetrate the leaf sheath around the same time on plants in different salinity levels. Total probing duration was 6 and 5% higher in control and low salinity plants, respectively than the high salinity plants. BPH spent less time in non-probing phase (NP) on the control and low salinity plants. Compared to control and low salinity TN1 plants, BPH on high salinity plants required 32 and 31 min, respectively more time to first phloem contact. BPH on high salinity plants spent significantly more time and took higher number of phloem salivating events prior to phloem sap ingestion than on the control and low salinity plants. BPH spent significantly less time in the phloem and sustained phloem sap ingestion was lower on high salinity plants. Additionally, BPH showed a significantly longer duration of xylem sap ingestion on plants in high salinity than on the control and low salinity plants. Planthoppers also started to penetrate the leaf sheath around same time on TPX plants at different salinity levels. Unlike on TN1, the total probing durations on TPX were 20 and 13% higher in the high salinity plants than on the control and low stressed plants, respectively. The lower numbers of NP phase and longer sustained phloem sap ingestion on high salinity TPX plants probably indicate the improvement in feeding efficiency.

### Effects of salinity stress on BPH salivary flange production

**Figure 4:**
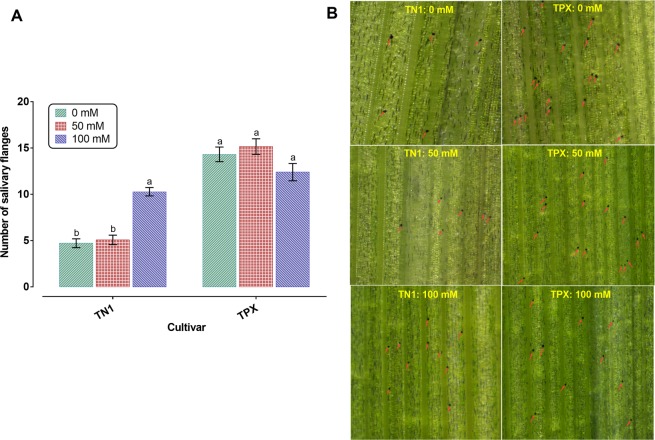
Effects of salinity stress on number of salivary flanges of BPH. (**A**) Salivary flanges produced by BPH on TN1 and TPX across different salinity levels. Each bar represents the mean ± SE from 25 replicates. Bars with different letters are significantly different among different salinity levels within a given cultivar (one-way ANOVA with a Tukey’s multiple comparison test, *P* < 0.05). (**B**) Representative replicates showing salivary flanges produced by BPH on rice plants at different salinity levels. Flanges are indicated by arrows. The figures were prepared and combined using Graphpad Prism version 8.3.1 for macOS (www.graphpad.com).

The number of salivary flanges of BPH in various salinity levels indicated that salinity stress induced significant increases in salivary flanges at high salinity TN1 plants compared to control and low salinity plants (*P* < 0.001). On the other hand, there was no significant difference in salivary flanges among the treatments of TPX plants (*P* = 0.073). BPH produced 13 and 18% lower salivary flanges on high salinity TPX plants than on control and low salinity plants, respectively (Fig. 4).

### Effects of salinity stress on BPH host preference

**Figure 5:**
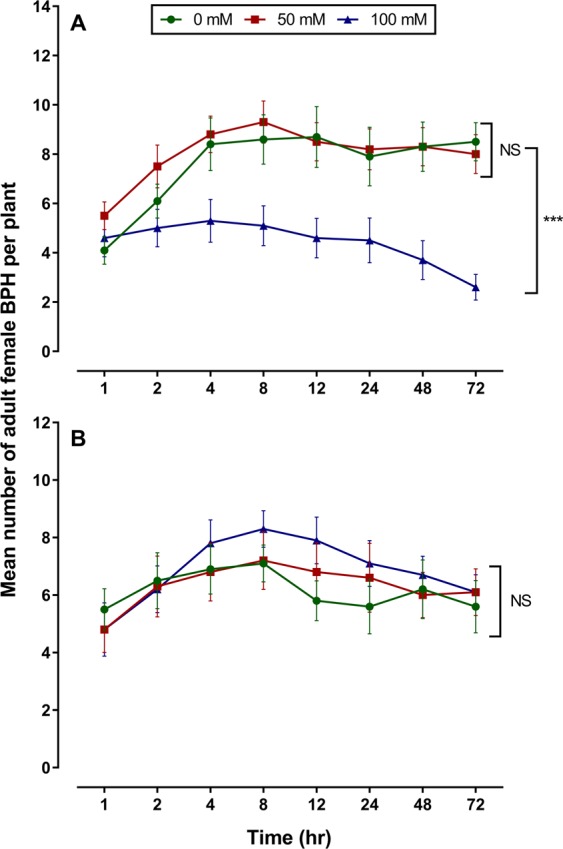
Host preference of BPH female by free choice studies on salt treated TN1 (**A**) and TPX (**B**) plants. Statistical results are shown as mean ± SEM (n = 10), ***P  <  0.001, based on two-way ANOVA. The figures were prepared and combined using Graphpad Prism version 8.3.1 for macOS (www.graphpad.com).

BPH showed a clear preference for the control and low salinity stressed TN1 plants, with the highest differences in the rate of settling being 1.58 and 1.66-folds greater than on high salinity plants at 08 h post infestation (*P* = 0.001), although significant differences already occurred 04 h post infestation (*P* = 0.002). However, there was no significant difference in BPH settling on TPX plants for all salinity levels (*P* = 0.233) (Fig. 5).

### Effects of salinity and BPH on ABA signaling pathway

**Figure 6:**
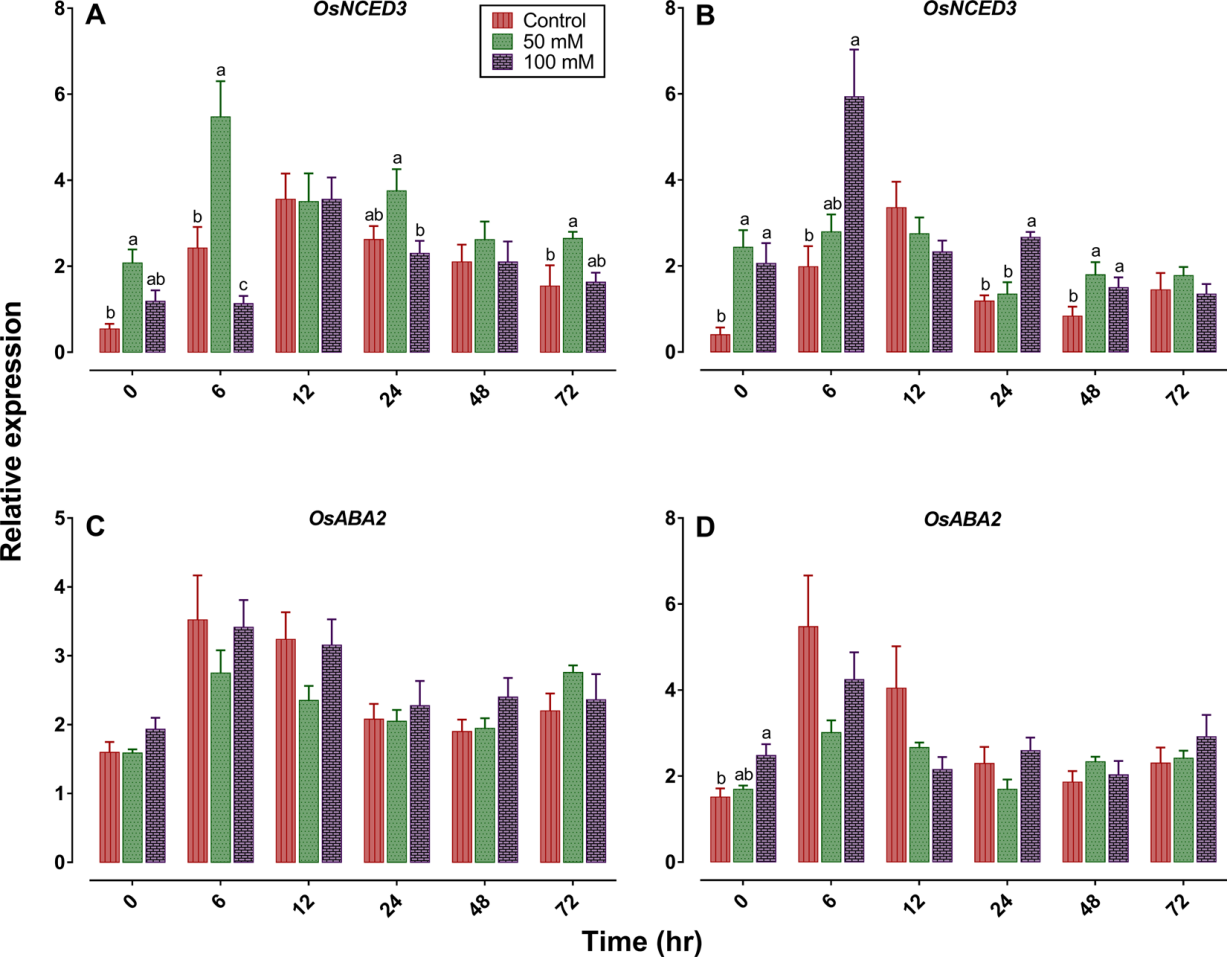
Differential expression levels of ABA biosynthesis-related genes in TN1 (**A,C**) and TPX (**B,D**) plants subject to salinity stress and BPH infestation. In all panels, mean and SE are based on four independent experiments. Bars with different letters are significantly different among different salinity levels within a given time (Tukey’s multiple range test, *P* = 0.05). The figures were prepared and combined using Graphpad Prism version 8.3.1 for macOS (www.graphpad.com).

Our RT-qPCR analysis revealed that the *OsNCED3* (*9-cis-epoxycarotenoid dioxygenase3*) expression levels was significantly lower in high salinity TN1 plants 6 hr post infestation compared to control and low salinity plants. In contrast, it was significantly higher in the high salinity TPX plants at 6, 24 and 48 h post infestation than on the control plants (Fig. 6A,B). No significant differences were detected in *OsABA2* (*Alcohol dehydrogenase2*) gene expression among different salinities in both TN1 and TPX plants (Fig. 6C,D). These results suggested that the increased expression of *OsNCED3*, might contribute in the higher ABA content in the high salinity TPX plant and lower ABA in high salt treated TN1 plants in response to BPH infestations.

### Effects of salinity on BPH induced defense gene expression

**Figure 7:**
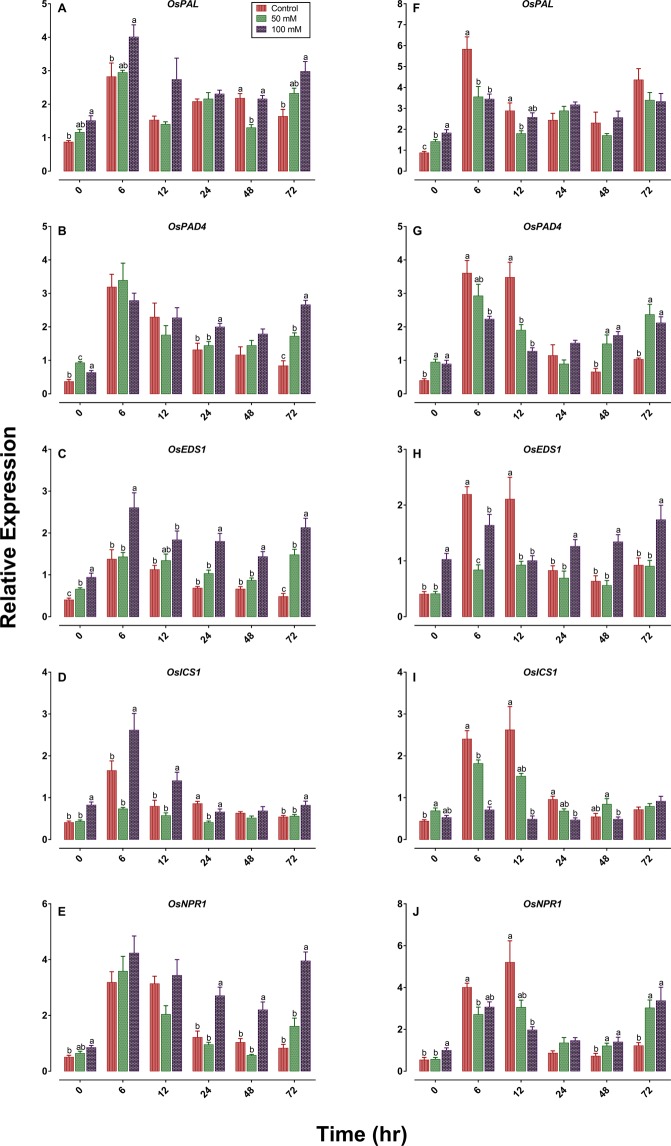
Differential expression of defense-related genes in TN1 (**A–E**) and TPX (**F–J**) plants response to salinity stress and planthopper infestation. *OsPAL*, *OsPAD4*, *OsEDS1* and *OsICS1* genes are involved in the SA-synthesis pathway. *OsNPR1* is a key regulatory gene in SA-dependent systemic acquired resistance. In all panels, mean and SE are based on four independent experiments. Bars with different letters are significantly different among different salinity levels within a given time (Tukey’s multiple range test, *P* = 0.05). The figures were prepared and combined using Graphpad Prism version 8.3.1 for macOS (www.graphpad.com).

Transcription of genes encoding the enzymes *OsPAL* (*phenylalanine ammonia-lyase*), *OsPAD4* (*phytoalexin deficient 4*), *OsEDS1* (*enhanced disease susceptibility 1*), *OsICS1* (*isochorismate synthase 1*) and *OsNPR1* (*Nonexpressor of pathogenesis-related genes1*) regulator factors induced by BPH infestation as part of the early defense response was significantly higher in high salinity plants than in the control and low salinity TN1 plants (Fig. 7A–E). In contrast, expression of these genes was significantly lower in the high salinity TPX plants compared to the control and low salinity plants (Fig. 7F–J). Up-and down-regulation of these genes in TN1 and TPX plants varied with post infestation times (Fig. 7).

## Discussion

We have identified a link between salinity stress, plant and herbivore fitness that have implications to our understanding of plant responses to biotic and abiotic stresses. Our results showed that salinity stress can have different consequences for plant resistance to BPH. We hypothesize that these consequences are a result of changes in plant defense response which is attributed to a variation in BPH feeding behavior.

In our study, planthopper infestation of TPX (moderately salt tolerant cultivar) plants under high salinity conditions led to higher losses in plant biomass, whereas the loss was lower in high salinity TN1 (salt sensitive cultivar) plants. Therefore, our plant bioassays indicated clear shifts in plant resistance of TPX and TN1 plants (decreasing and increasing on salinity stressed plants, respectively) in response to BPH feeding. Further, the changes in plant resistance that we noted under high salinity, appreciably translated into lower and higher BPH densities on high salinity TN1 and TPX cultivars, respectively. This cultivar-dependent response may be attributed to variation in morphological and metabolic changes of the cultivars to salt stress^47^, which consequently affect the herbivore performance. Verdugo *et al*.^48^ also reported that herbivore performance varied on susceptible and resistant varieties under stress condition.

The accumulation of metabolites in the plant is regarded as a common response to different abiotic stresses^49^. The osmotic adjustment in the plant can be achieved by this accumulation of sugars, sugar alcohols, and amino acids^50–52^. Furthermore, different sensitivity of cultivars to salt showed different metabolite changes^53^. Therefore, we hypothesized that changes in host nutritional quality consequently affect the feeding behavior of BPH. EPG data showed that BPH spent the least amount of time feeding from the phloem on high salinity TN1 plants and thus reduced population growth of BPH. In contrast, BPH tended to spend more phloem sap ingestion time in the high salinity stressed TPX plants and their populations grew significantly higher. Like EPG, differential impact was also reflected in BPH feeding behavior on different cultivars as determined by honeydew production, salivary flange production and host preference studies. It is understood that BPH improved feeding efficiency in high salinity TPX might be due to increased nitrogenous compound content in plant under salinity stress. However, inferior feeding efficiency in salinity stressed TN1 plants indicated that other changes might outweigh the benefits to the BPH from increased metabolite content on salinity-stressed plants. It is plausible that in addition to changes in nutrition, salinity-induced osmotic stress varied with salt-resistant and sensitive cultivars which may have different consequences in nutrient absorption. Foster and Treherne^54^ reported that salinity-induced osmotic stress negatively impacted the absorption of essential sugars by herbivores from host tissues. Since, the accumulation of osmo-potential metabolites such as mannitol and trehalose can drastically reduce in salt sensitive rice variety than in salt tolerant varieties^47^ and these metabolites can decrease osmotic potentials of the cytoplasm in shoot/root cells to avoid cell dehydration under salt stress^55^. A possible explanation could be that the lower accumulation of these metabolites might be contributed to cell dehydration in salt-sensitive varieties, which therefore can hinder the BPH nutrient absorption from host tissues.

Typically, ABA is crucial to protect the plant from abiotic stresses such as drought, salinity, cold, heat stress and wounding^56,57^; however, its exact role in response to herbivore is still quite unknown. *OsNCED3* and *OsABA2* genes have a vital role to play in ABA biosynthesis during drought and salt stress in rice^58–61^. The induction of ABA genes, *OsNCED3* and *OsABA2* before BPH infestation introduction clearly indicates the importance of ABA under salinity stress. The up-regulation *OsNCED3* gene in salinity stressed TPX plants and down-regulation of the same gene in high salinity TN1 plant compared to control plants after BPH infestation indicate that plant response is not unique under biotic and abiotic stress, largely cultivar dependent. Guo *et al*.^30^ also found that ABA signaling gene expression varied with plant genotype in response to aphid feeding under stress conditions. The ABA signaling pathway under salinity stress could affect the BPH induced defense responses by altering the SA signaling pathways. In our study, SA signaling genes significantly increased in response to BPH infestation in the high salinity TN1 plants and decreased in the high salinity TPX plants. A corresponding decrease in ABA gene expression in high salinity TN1 plant and increase in high salinity TPX plants clearly indicate the antagonism between ABA and SA. Therefore, our study showed ABA and SA antagonism as an important factor in the interaction of salinity stress and BPH (Supplementary Fig. S1). The results thus corroborate with previous studies reporting that hormonal crosstalk under multiple stresses is indispensable to enable plants to coordinate and prioritize their reactions for survival with limited resources^62,63^.

In conclusion, our study demonstrated that salinity stress markedly shaped the plant-herbivore interactions (Supplementary Table S3). Plant biomass bioassay clearly indicated the variation in ontogenetic changes in plant tolerance to BPH infestation under salinity stress. Population index studies showed cultivar dependent consequences of BPH under salinity stress. We also showed that different consequences associated with BPH feeding on corresponding plants. Our study suggests that salinity stress is involved in changes of different hormonal signaling. ABA and SA antagonism is a key element to reveal the mechanism of BPH interactions to salinity stressed plants. A comprehensive study including phytohormones analysis and transcriptomic, proteomic and metabolomics approaches might provide further understanding of plant-insect interactions under salinity stress.

## Materials and methods

### Plant growth conditions

Rice *indica* cultivars, Taichung Native1 (TN1), IR64, HongHai Dao (HHD) and TaiPing Xian (TPX) were used in the experiments. TN1 and IR64 seeds were imported from International Rice Research Institute, Los Baños, Philippines. HHD and TPX seeds were collected from Guangxi Academy of Agricultural Sciences and China National Rice Research Institute, respectively. TN1 is highly susceptible to BPH and sensitive to salinity. The cultivar IR64 moderately salt-tolerant, is moderately resistant to BPH. HHD is moderately salt-tolerant; but susceptible to BPH. TPX is moderately salt-tolerant and resistant to BPH (Supplementary Table S4 and S5). The seedlings were grown into a cultivation box (50 × 40 × 10 cm) filled with the nutrient solution^64^ and placed in a control chamber set at 27 ± 1 °C, 16:8 h light:dark photoperiod and photosynthetic photon flux density of 650–700 μmol m^−2^ s^−1^. To avoid anoxia, nutrient solution kept aerated by aquarium pump. The pH of nutrient solution was adjusted to 5.0 using 1 N KOH/HCl twice a day and renewed every three days. 25-days-old seedlings were used in the experiment.

### Insect rearing

BPH adults and nymphs used in the study were originally collected from a field population in the Huajiachi campus of Zhejiang University, Hangzhou, China. The insects were reared on susceptible rice seedlings cv. Taichung Native 1 (TN1) at 26 ± 1 °C, 70 ± 5% relative humidity and a 16:8 h light:dark photoperiod.

### Salt stress treatment

We recently reported the bottom-up effects of salinity stress on population parameters of the BPH at a series of salinity levels^16^. Based on this study the experimental treatments consisted of three salinity levels (0, 50 and 100 mM). 25-days-old hydroponically grown seedlings were subjected to different salinized nutrient solution. To prepare the plant, cultivation box (50 × 40 × 10 cm) was filled with 24 L of nutrient solution (control) or salinized nutrient solution. The salinity treatments were created by adding NaCl and CaCl_2_ (AR grade) (2:1 molar concentration). The salinity measurement verified by an EC meter (5 dS m^−1^ for 50 mM and 10 dS m^−1^ for 100 mM salinity level). The plants were allowed to grow in salinized solution for five days prior to introduction of herbivores.

### Plant bioassay study

The experimental design was 4 × 3 × 2 factorial with ten independent biological replications which consisted of four cultivars (TN1, IR64, HHD and TPX), three salinity levels (0, 50 and 100 mM) and two planthopper infestations (uninfested and infested). One seedling of each cultivar was placed in black plastic cup (d × h = 10 × 12 cm) contained 400 mL of the nutrient or salinized nutrient solution which was covered with a well-ventilated transparent plastic container. Twenty-five 2^nd^-3^rd^ instar hopper nymphs were introduced onto each plant. Similar set of uninfested plants were maintained. Plants were monitored daily and the solution was replaced in every three days. When the plants of susceptible cultivar started to wilt (after 6 days), nymphs were collected, oven dried for 48 h and weighed. Infested and uninfested plants of each treatment were collected, oven-dried for 72 h and weighed. Plant biomass losses at different salinity levels were calculated through comparing the dry weight of planthopper infested and uninfested plant of same salinity level. Functional plant loss index was determined by using the following formula^65^:

$${\rm{Functional}}\,{\rm{plant}}\,{\rm{loss}}\,{\rm{index}}({\rm{FPL}}, \% )=\left(1-\frac{{\rm{Dry}}\,{\rm{weight}}\,{\rm{of}}\,{\rm{infested}}\,{\rm{plant}}}{{\rm{Dry}}\,{\rm{weight}}\,{\rm{of}}\,{\rm{uninfested}}\,{\rm{plant}}}\right)\times 100$$

### BPH population growth

Population growth study was conducted using the method described by Ge *et al*.^66^ with some modifications. The experiment was set with four cultivars (TN1, IR64, HHD and TPX) and three salinity levels (0, 50 and 100 mM). The study was arranged with a randomized complete block design with five replications. Two pair of newly emerged BPH was introduced to seedling settled in a black plastic cup containing 400 ml nutrient or salinized nutrient solution. The cup was then covered with a transparent well-ventilated plastic container. After three days planthoppers were transferred to a new set of treated seedlings. Then seedlings in old cups were checked daily for newly hatched nymphs. When no newly hatched nymphs were found after a 72 h period, the seedlings were removed and dissected under a stereomicroscope (Nikon C-LEDS) to check for inviable eggs. Nymphs of the new generation were counted and transferred into another new plastic cup with the same aged treated rice plants. These steps were continued until the original female died. Nymphs in the new plastic were checked in every two days until adult emergence and adult number was recorded. The plants and solution of these cups were replaced in every three days. The population growth index (PGI) was calculated by the ratio of N_1_/N_0_, where N_1_ is the total number of adult and N_0_ is the number of adults introduced initially (N_0_ = 4).

### Honeydew production

Honeydew excretion is an important indicator for assessing feeding behavior of Homopteran pests^67^. Among the different techniques, filter paper^68^ and parafilm sachet method^69^ are widely used to measure the feeding response of BPH. In our study, both techniques were used to examine the honeydew production of BPH female on TN1, IR64, HHD and TPX cultivars under different salinity levels (0, 50 and 100 mM). In parafilm sachet method, pre-weighed parafilm bag (Pechiney Plastic Packaging Company, Chicago, IL) was attached with plant at about 6–8 cm above the root. One newly emerged pre-starved (24 h) female was placed into each bag. After 24 hr feeding, the female was removed from the parafilm bag and the bag with honeydew was weighed using a Mettler Toledo balance (Model: XS105DU, Switzerland, d = 0.01 mg). The females used in the study were collected, oven-dried at 60 °C for 3 days, and weighed to standardize honeydew production by each BPH. The bioassay was replicated 6 × (n = 5) times in a completely randomized block design. In filter paper method, seedlings of each treatment were infested with two newly emerged pre-starved (24 hr) females in specially prepared glass chamber. The glass chamber allowed the females to feed within 5 cm of the plant base and placed over filter paper, precisely fixed around the seedling shoot. The filter paper was treated with bromocresol green (2 mg/mL of 70% ethanol) to point out the honeydew type as coming from phloem (blue rimmed spots) or xylem (white spots). After 24 hr feeding, the excreted honeydew areas on filter paper were measured using Image-J software (National Institute of Health, USA). The weight of insects used in the bioassay was measured as stated above and used in the standardization of honeydew production. The bioassay was laid out in completely randomized block design with ten replications.

### EPG recording

Salinity stress showed no significant difference on plant-insect interactions in the case of HHD, and IR64 followed a similar pattern as TN1 in plant bioassay, population growth index and honeydew production. Therefore, we selected TN1 and TPX for further studies. EPG technique was employed to observe the feeding behavior of BPH on TN1 and TPX cultivars at 0, 50 and 100 mM salinity levels. A Giga-8 direct current electrical penetration graph (DC-EPG) amplifier system with a 10^9^-Ω input resistance in a Faraday cage (manufactured by Wageningen University, Wageningen, The Netherlands) was used for this observation^70^. Newly emerged macropterous females were starved for 2 hr prior to use in experiment. A gold wire (20 μm in diameter) was attached on the dorsum of the anesthetized insect with water soluble conductive silver glue, while other end of the wire was connected to the amplifier through a copper nail that was inserted into the EPG probe. The wired BPH then gently placed on rice leaf sheath of plant. A copper wire (2 mm diameter × 10 cm length) connected to the amplifier was vertically inserted into the solution act as plant electrode. Planthoppers were then allowed to feed on rice plant for 6 hours. The EPGs were recorded in a quiet room with ambient conditions of 26 ± 2 °C, RH 70 ± 5% and constant light for 6 h. A different experimental insects and plants were used for each replicate. The experiment was repeated until 10 biological replications of each treatment were obtained. The EPG data were analyzed using the Stylet+ software (Wageningen Agricultural University, Netherlands) to distinguish various waveforms. EPG signals were classified into five different waveforms according to the relative voltage level^71^ (Supplementary Fig. S2).

### Salivary flange quantification

Salivary flanges of BPH on TN1 and TPX cultivars at 0, 50 and 100 mM salinity levels were quantified according to Cao *et al*.^72^. One newly emerged macropterous female (pre-starved for 2 h) was introduced to seedlings of each treatment in specially prepared glass chamber. The glass chamber confined the females within 5 cm of the plant base. After 24 h feeding, insect was removed from the plant and leaf sheath was cut from the base, immediately immersed into 0.1% crystal violet for 10–15 minutes. The number of salivary flanges was then counted under a stereo microscope (Nikon, model: C-LEDS, China). The experiment was replicated 25 times in a completely randomized block design.

### Host plant free-choice behavior

Host preference is an important insect phenomenon by which they look for suitable resources to get food, oviposit and set up nesting sites. Host plant preference of BPH females among plants treated with different salinity levels (0, 50 and 100 mM) was observed using TN1 and TPX cultivars in accordance to Tan *et al*.^73^. Three glass tubes with one seedling (2 salinity treated along with control) were confined in a well-ventilated transparent plastic chamber (d × h = 10 × 45 cm). Twenty (25) macropterous females were placed in the center among three seedlings. The numbers of females settled on each plant were counted after 1, 2, 4, 8, 12, 24, 48 and 72 h of insect release. The study was replicated 10 times on a plant chamber set at 27 ± 1 °C and 16:8 h light:dark photoperiod.

### Gene expression analysis

Seedlings of TN1 and TPX at 0, 50 and 100 mM salinity levels were infested with twenty five 2^nd^-3^rd^ instar nymphs. The leaf sheath (feeding site) of each treatment was collected at 0, 6, 12, 24, 48 and 72 h post infestation and stored at −80 °C until further use. Total RNA was extracted from 100 mg of rice sample using TRizol reagent (Invitrogen) according to manufacturer’s instructions. The quantity of RNA was determined by measuring OD_260_ nm with the NanoDrop2000 Spectrophotometer (Thermo Scientific). RNA was reversely transcribed to cDNA using ReverTra ACE qPCR RT Master Mix with gDNA remover (Toyobo, Japan). Quantitative RT-PCR was performed on a Bio-Rad CFX96 Real-Time System (Bio-Rad Laboratories, Hercules, CA). qPCR reaction mixtures contained 2 µl of diluted cDNA as template, 10 µl of SYBR Green Real-time PCR Master Mix (Toyobo, Japan), 6 µl of ddH_2_O and 1 µl of each gene-specific primer in a total volume of 20 µl. The thermal profile was one cycle of 95 °C for 3 min followed by 40 cycles of 95 °C for 10 s, and 60 °C for 30 s. The specificity of all listed primers (Supplementary Table S6) was confirmed with melting curves. The *OsActin gene* was used as an internal standard to normalize cDNA concentration^46^. Four biological replicates were used for all qPCR measurements. We transferred the fold changes to Log_2_ before statistical analysis to normalize data. Nevertheless, differential gene expression in the figure was calculated from the original data.

### Statistical analyses

We verified all data for normality and homogeneity of variance by Levene’s test prior to perform an analysis of variance (ANOVA). Two-way ANOVA, followed by Tukey’s post hoc test for multiple comparisons were performed for plant bioassays, BPH population growth rates, honeydew production, and host preference. Functional plant loss percentage, proportion of xylem-honeydew excretion data were subjected to arcsine square root transformation prior to ANOVA. But the data in the figure were calculated from original data. The EPG data were analyzed by non-parametric Kruskal-Wallis test followed by a separate pair-wise comparison using Mann-Whitney *U*-test (*α* = 0.05). Planthopper salivary flange production and plant genes expression over time was analyzed using one-way ANOVA followed by Tukey’s post hoc test. All data were analyzed using SPSS 16.0 (SPSS Inc, New York, USA).

### Compliance with ethical standards

#### Ethical approval

This article does not contain any studies with human participants or animals performed by any of the authors.

## Supplementary information

Supplementary information.

## Data Availability

All data generated or analysed during this study are included in this published article (and its Supplementary Information file).
