# Supplementary material for: Interactions between brown planthopper (Nilaparvata lugens) and salinity stressed rice (Oryza sativa) plant are cultivar-specific
Source: Sci Rep. 2020 May 15;10:8051. doi: 10.1038/s41598-020-64925-1 (PMC7229203; doi:10.1038/s41598-020-64925-1)
Supplement: Supplementary file 1 — Supplementary information. [file 41598_2020_64925_MOESM1_ESM.docx]

**Supplementary information**

**Title:** Interactions between brown planthopper (*Nilaparvata lugens*) and salinity stressed rice (*Oryza sativa*) plant are cultivar-specific

**Authors:** Md Khairul Quais^1, 2^, Asim Munawar^1^, Naved Ahmad Ansari^1^, Wen-Wu Zhou^1^, Zeng-Rong Zhu^1*^

**Affiliation:**^1^State Key Laboratory of Rice Biology, Ministry of Agriculture; Key Laboratory of Molecular Biology of Crop Pathogens and Insects; Institute of Insect Sciences, Zhejiang University, Hangzhou, Zhejiang, 310058, China

^2^Senior Scientific Officer, Rice Farming Systems Division, Bangladesh Rice Research Institute, Gazipur, Bangladesh

***Corresponding author:**

Zeng-Rong Zhu, Ph.D., Email: [zrzhu@zju.edu.cn](mailto:zrzhu@zju.edu.cn)

**Journal name:** Scientific Reports

***Content***

**Supplementary Table S1.** Growth parameters of rice cultivars in response to planthopper challenge under different salinity levels

**Supplementary Table S2.** Electrically recorded feeding parameters of BPH females on salt treated rice plants

**Supplementary Table S3.** Summary of the salinity induced impact on plant-insect interactions

**Supplementary Table S4.** Reaction of tested rice cultivars in terms of modified standard evaluation score (SES) under salinity

**Supplementary Table S5.** Reaction of different rice cultivars to brown plant hopper under standard seedbox screening technique

**Supplementary Table S6.** Forward (F) and reverse (R) primer sequences used in real-time quantitative PCR

**Supplementary Figure S1.** A model summarizing the ABA-SA antagonism under biotic and abiotic stress

**Supplementary Figure S2.** Typical EPG waveforms identified from BPH feeding on rice plants

**Supplementary Table S1.** Growth parameters of rice cultivars in response to planthopper challenge under different salinity levels

| **Cultivar** | **Salinity level (mM)** | **Insect infestation** | **Shoot length (cm)^a^** | **Root length (cm) ^a^** | **Shoot dry wt. (g) ^a^** | **Root dry wt. (g) ^a^** |
| --- | --- | --- | --- | --- | --- | --- |
| TN1 | 0 | Control | 53.32±1.69 | 8.95±0.27 | 0.439±0.02 | 0.064±0.004 |
|  |  | Infested | 45.55±0.43 | 8.50±0.31 | 0.197±0.01 | 0.034±0.002 |
|  | 50 | Control | 44.34±0.66 | 8.68±0.45 | 0.369±0.02 | 0.051±0.002 |
|  |  | Infested | 44.50±0.64 | 7.96±0.24 | 0.144±0.01 | 0.036±0.001 |
|  | 100 | Control | 43.44±0.38 | 8.85±0.30 | 0.156±0.01 | 0.039±0.002 |
|  |  | Infested | 39.76±0.73 | 7.29±0.20 | 0.097±0.01 | 0.022±0.001 |
| IR64 | 0 | Control | 58.26±0.78 | 13.90±0.16 | 0.491±0.01 | 0.145±0.007 |
|  |  | Infested | 53.96±0.73 | 12.94±0.20 | 0.408±0.02 | 0.091±0.005 |
|  | 50 | Control | 47.94±0.75 | 12.58±0.19 | 0.299±0.02 | 0.092±0.002 |
|  |  | Infested | 47.24±0.71 | 11.62±0.31 | 0.228±0.01 | 0.057±0.001 |
|  | 100 | Control | 46.38±0.64 | 12.07±0.38 | 0.236±0.01 | 0.068±0.002 |
|  |  | Infested | 43.98±0.40 | 11.68±0.33 | 0.181±0.01 | 0.041±0.002 |
| HHD | 0 | Control | 64.22±1.21 | 13.48±0.41 | 0.534±0.03 | 0.150±0.012 |
|  |  | Infested | 54.96±0.85 | 13.04±0.34 | 0.431±0.01 | 0.083±0.005 |
|  | 50 | Control | 49.46±1.01 | 14.10±0.43 | 0.337±0.02 | 0.087±0.006 |
|  |  | Infested | 48.70±0.73 | 13.30±0.38 | 0.273±0.01 | 0.061±0.003 |
|  | 100 | Control | 47.64±0.53 | 12.90±0.28 | 0.279±0.02 | 0.082±0.013 |
|  |  | Infested | 46.30±0.60 | 12.08±0.16 | 0.209±0.01 | 0.052±0.011 |
| TPX | 0 | Control | 61.07±1.16 | 12.28±0.48 | 0.468±0.03 | 0.162±0.019 |
|  |  | Infested | 61.18±0.72 | 12.40±0.15 | 0.473±0.02 | 0.117±0.006 |
|  | 50 | Control | 50.80±0.65 | 13.30±0.55 | 0.293±0.02 | 0.086±0.008 |
|  |  | Infested | 48.32±0.70 | 11.02±0.22 | 0.255±0.01 | 0.055±0.004 |
|  | 100 | Control | 48.04±0.82 | 11.30±0.21 | 0.269±0.01 | 0.070±0.006 |
|  |  | Infested | 44.71±0.73 | 11.01±0.38 | 0.165±0.01 | 0.046±0.004 |
| *F*-Cultivar (C)^b^ | | | 99.39*** | 257.74*** | 49.50*** | 65.71*** |
| *F*-Salinity (S) ^b^ | | | 447.30*** | 21.07*** | 407.97*** | 132.17*** |
| *F*- Insect infestation (I) ^b^ | | | 81.50*** | 36.05*** | 186.80*** | 141.46*** |
| *F*- C×S ^b^ | | | 8.70*** | 3.03** | 7.38*** | 9.92*** |
| *F*- C×I ^b^ | | | 2.06^NS^ | 0.12^NS^ | 18.03*** | 2.72* |
| *F*-S×I ^b^ | | | 14.74*** | 2.74^NS^ | 2.35^NS^ | 7.84*** |
| *F*- C×S×I ^b^ | | | 6.50*** | 2.47* | 7.70*** | 0.48^NS^ |

Nominator df =3 (Cultivar), 2 (Salinity), 1 (infested/control), 6 (C×S), 3 (C × I), 2 (S× I), 6 (C× S× I); denominator df =216.
^a^ Mean ± standard error (N= 10).
^b^ NS= *P*> 0.05, * = *P* ≤ 0.05, ** = *P* ≤ 0.01, *** = *P* ≤ 0.001.

**SupplementaryTable S2.** Electrically recorded feeding parameters of BPH females on salt treated rice plants

| **EPG parameter** | **TN1** | | | **TPX** | | | |
| --- | --- | --- | --- | --- | --- | --- | --- |
|  | **0 mM** | **50 mM** | **100 mM** | | **0 mM** | **50 mM** | **100 mM** |
| Time to first probe (min) | 0.8±0.3a | 1.3±0.5a | 1.1±0.3a | | 4.6±2.1a | 6.9±4.0a | 4.0±2.9a |
| Probing duration (min) | 358.3±0.7a | 356.8±1.8ab | 337.8±9.7b | | 209.2±12.3b | 229.7±21.0ab | 263.1±11.1a |
| Time from 1^st^ probe to 1^st^ phloem contact (min) | 21.1±3.7b | 22.1±4.8b | 52.8±11.6a | | 100.7±17.1a | 78.2±26.0a | 68.7±14.4a |
| Number of phloem salivation event | 3.6±0.8b | 5.9±1.2b | 10.0±1.4a | | 7.5±1.4a | 9.2±2.3a | 9.4±2.1a |
| Average duration of phloem salivation (min) | 8.5±2.4a | 5.9±1.6a | 6.3±1.4a | | 2.6±0.8a | 3.8±0.9a | 6.1±1.3a |
| Number of phloem ingestion event | 2.5±0.4a | 3.2±0.6a | 4.0±0.8a | | 1.8±0.5a | 2.9±0.9a | 3.3±0.8a |
| Average duration of phloem ingestion (min) | 157.7±30.7a | 124.8±30.5a | 35.3±6.6b | | 3.8±1.9b | 13.3±4.6ab | 28.6±8.1a |
| No. of sustained phloem ingestion (>10 min) | 1.9±0.3a | 2.2±0.3a | 2.0±0.4a | | 0.2±0.1b | 0.8±0.3ab | 1.5±0.3a |
| Avg. duration of sustained phloem ingestion | 210.4±27.3a | 143.4±26.2a | 60.4±12.0b | | 5.2±3.1b | 18.1±6.1ab | 37.9±8.0a |
| Number of xylem events | 4.0±1.0a | 6.0±1.2a | 7.1±1.3a | | 4.3±1.3a | 6.5±2.3a | 7.9±1.6a |
| Average duration of xylem ingestion (min) | 3.5±1.3a | 4.6±1.0a | 7.9±3.3a | | 7.0±4.2a | 8.4±3.5a | 7.3±1.4a |

Values represent means ± SEs from 10-13 replications. Different letters indicate significant difference among different salinity levels within a given cultivar (*α* = 0.05)

**Supplementary Table S3.** Summary of the salinity induced impact on plant-insect interactions

Red and green arrows indicate negative and positive effects, respectively, while the arrow thickness indicates the strength of interaction

- **Evaluation of rice varieties under saline condition**

**Supplementary Table S4.** Reaction of tested rice cultivars in terms of modified standard evaluation score (SES) under salinity stress

| **Variety** | **Salinity score** | **Reaction to salinity** |
| --- | --- | --- |
| TN1 | 7.23 | Susceptible |
| IR64 | 5.50 | Moderately tolerant |
| Hong Hai Dao (HHD) | 5.21 | Moderately tolerant |
| Taiping Xian (TPX) | 4.75 | Moderately tolerant |

Seedlings were challenged with salinity stress (100 mM) at 14 days after emergence. SES was recorded on 12 days after salt treatment with a score 1 for tolerant and 9 for sensitive^1^

Assessment scores of seedlings with respect to relative salt tolerance

| **Score** | **Visual observation** | **Relative tolerance** |
| --- | --- | --- |
| 1 | Normal growth; no leaf symptoms | Highly tolerant |
| 3 | Nearly normal growth; but occasional white leaf tips and rolled leaves | Tolerant |
| 5 | Growth severely retarded; most leaves rolled, few leaves elongate | Moderately tolerant |
| 7 | Complete cessation of growth; most leaves dry and some seedling death | Susceptible |
| 9 | Most seedling dead or dying | Highly susceptible |

Source: Adapted from Bado *et al.* (2016)^1^

- **Evaluation of rice genotypes for brown planthopper (BPH) resistance**

**Supplementary Table S5.** Reaction of different rice cultivars to brown plant hopper under standard seedbox screening technique

| **Variety** | **Damage score** | **Reaction to BPH** |
| --- | --- | --- |
| TN1 | 9 | Susceptible |
| IR64 | 5 | Moderately resistant |
| Hong Hai Dao (HHD) | 7 | Susceptible |
| Taiping Xian (TPX) | 1 | Resistant |

Interpretation of results was done with a mean rating of 0 to 3, 3.1 to 6.9 and 7 to 9 designated as resistant, moderately resistant and susceptible, respectively^2^

Assessment scores of seedlings with respect to BPH tolerance

| **Score** | **Visual observation** |
| --- | --- |
| 0 | No injury |
| 1 | Very slight injury |
| 3 | First and 2nd leaves of most plants partially yellowing |
| 5 | Pronounced yellowing and stunting or about 10 to 25% of the plants wilting or dead and remaining plants severely stunted or dying |
| 7 | More than half of the plants wilting or dead and remaining plants severely stunted or dying |
| 9 | All plants dead |

Source: Adapted from IRRI (2013)^2^

**Supplementary Table S6.** Forward (F) and reverse (R) primer sequences used in real-time quantitative PCR

| **Gene** | **Accession no.** | **Primer sequences (5′→3′)** | **Reference** |
| --- | --- | --- | --- |
| *OsNCED3* | AY838899 | F: CCCCTCCCAAACCATCCAAACCGA | Zhu *et al*. (2009)^3^ |
|  |  | R: TGTGAGCATATCCTGGCGTCGTGA |  |
| *OsABA2* | MG334011 | F: TGTGGATCTGCTACCTAAGG | Jiang *et al*. (2019)^4^ |
|  |  | R: GTAAAGCCACCATCCACCATG |  |
| *OsPAL* | X16099 | F: CAAGAAGGTGCTCACCATGA | Hao *et al*. (2011)^5^ |
|  |  | R: CTTCTGCATGAGCGGGTAGT |  |
| *OsPAD4* | AK243523 | F: CCAACATGTACCGCATCAAG | Lu *et al*. (2018)^6^ |
|  |  | R: TGTCAAGTGGCTCAACAAGG |  |
| *OsEDS1* | AK100117 | F: CATTCCAAGAACGAGGACACTG | Lu *et al*. (2018)^6^ |
|  |  | R: CAAGACTCAAGGCTAGAACCGA |  |
| *OsICS1* | AK120689 | F: TTGAATGGGAGGAATTTGGA | Lu *et al*. (2018)^6^ |
|  |  | R: GATTGGAGCTCCTTCACAGC |  |
| *OsNPR1* | AK120715 | F: GGGAGAAAAGCGGTTCAAAT | Lu *et al*. (2018)^6^ |
|  |  | R: TGATGCTTCTCCGAGTTGTG |  |
| *OsActin* | AB047313 | F: GTCCTCTTCCAGCCTTCCTT | Lu *et al*. (2018)^6^ |
|  |  | R: GCAATGCCAGGGAACATAGT |  |

**
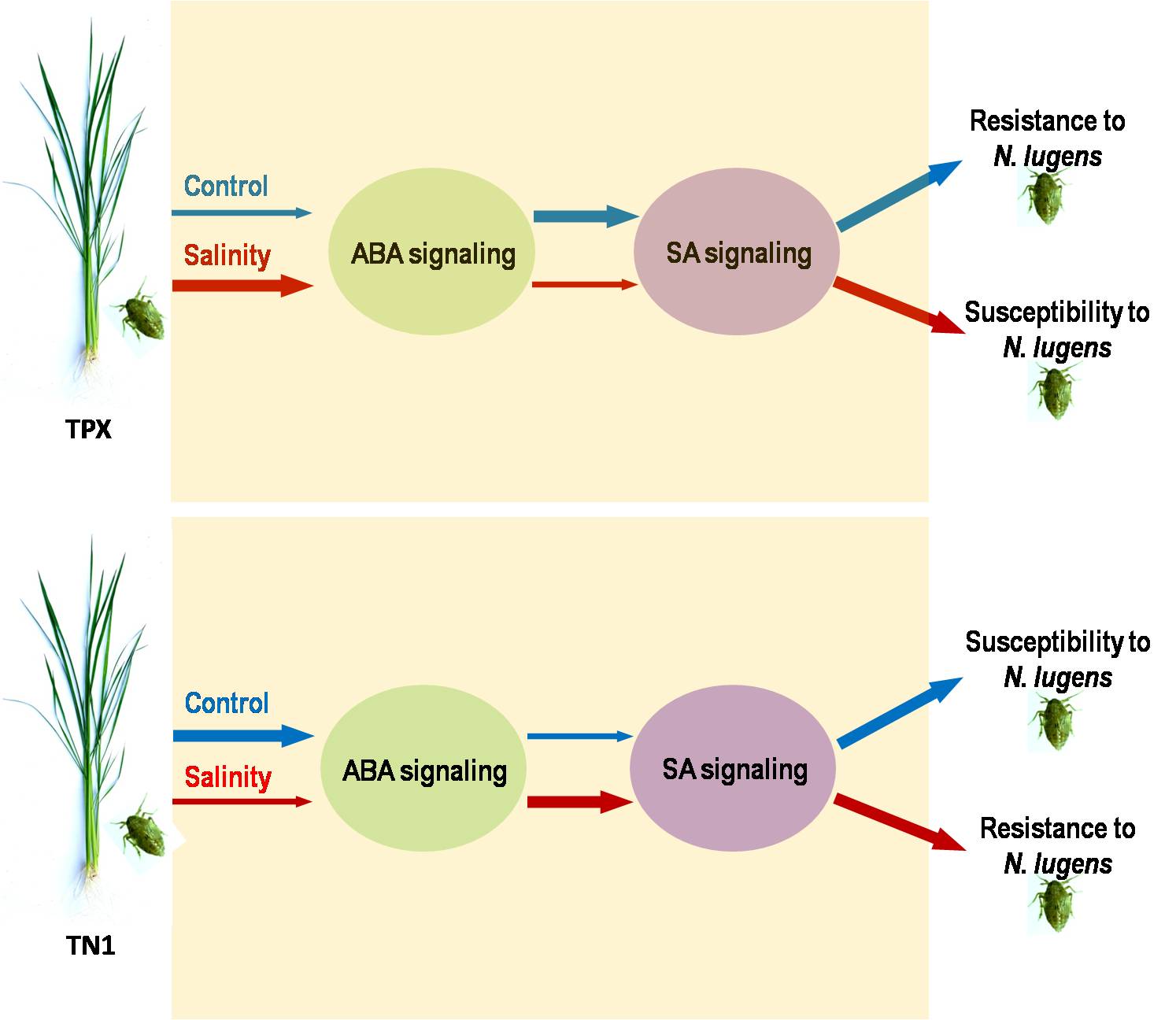
**

**Supplementary Figure S1.** A model summarizing the ABA-SA antagonism in TN1 and TPX rice plants under biotic and abiotic stress. Blue and red colored arrows indicate the interactions under control and high salinity conditions, respectively. Filling amount of arrow indicates the strength of the interactions. The figure was prepared using Graphpad Prism version 8.3.1 for macOS ([www.graphpad.com](http://www.graphpad.com/)).


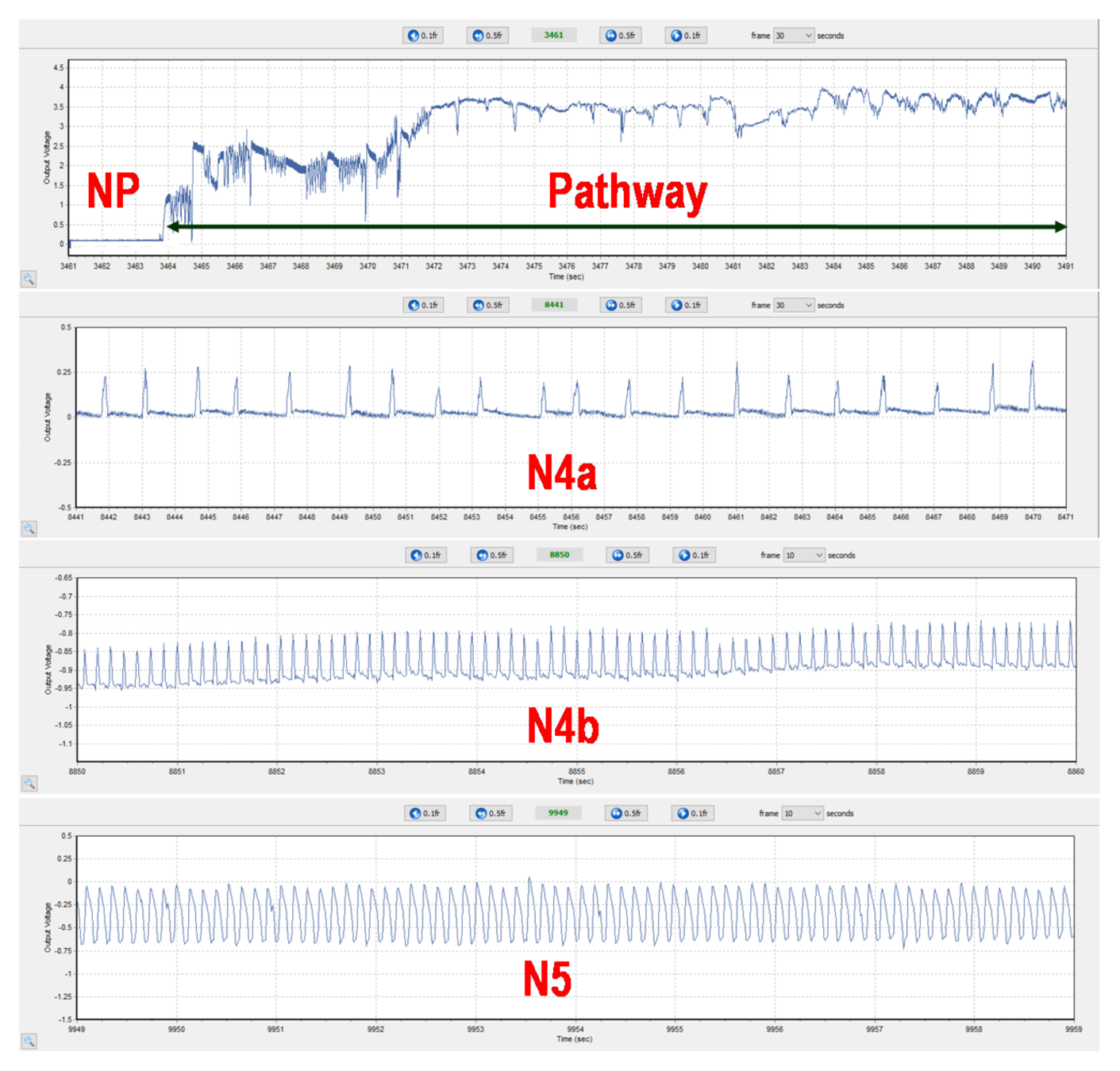


**Supplementary Figure S2.** Typical EPG waveforms identified from BPH feeding on rice plants. NP indicates non-penetration, Pathway comprises of penetration initiation, salivation and stylet movement and extracellular stylet activity near phloem region, N4a points phloem salivation, N4b phloem sap ingestion, and N5 xylem sap ingestion. The figures were combined using Graphpad Prism version 8.3.1 for macOS ([www.graphpad.com](http://www.graphpad.com/)).

**Supplementary references**

1 Bado, S. *et al.* Protocol for Screening for Salt Tolerance in Rice. In:Bado S. *et al.Protocols for pre-field screening of mutants for salt tolerance in Rice, Wheat and Barley.* Springer, Cham:21-31 (2016).

2 IRRI. Standard evaluation system for rice (SES). 5^th^ Edition. International Rice Research Institute, LosBanos, Phillipines (2013).

3 Zhu, G. H., Ye, N. H. & Zhang, J. H. Glucose-induced delay of seed germination in rice is mediated by the suppression of ABA catabolism rather than an enhancement of ABA biosynthesis. *Plant Cell Physiol.***50**, 644-651 (2009).

4 Jiang, D. G. *et al.* Overexpression of a microRNA-targeted NAC transcription factor improves drought and salt tolerance in rice via ABA-mediated pathways. *Rice***12**, 76 (2019).

5 Hao, Z. N., Wang, L. P., He, Y. P., Liang, J. G. & Tao, R. X. Expression of defense genes and activities of antioxidant enzymes in rice resistance to rice stripe virus and small brown planthopper. *Plant Physiol. Bioch.***49**, 744-751 (2011).

6 Lu, H. P. *et al.* Resistance of rice to insect pests mediated by suppression of serotonin biosynthesis. *Nat. Plants***4**, 338-344 (2018).
